# Preparation and Characterization of Di-, Tri-, and Tetranuclear Schiff Base Complexes Derived from Diamines and 3,4-Dihydroxybenzaldehyde

**DOI:** 10.1155/2013/219356

**Published:** 2013-12-25

**Authors:** Ahlam Jameel Abdulghani, Asmaa Mohammed Noori Khaleel

**Affiliations:** Department of Chemistry, College of Science, University of Baghdad, Jaderiya, Baghdad, Iraq

## Abstract

A series of new di-, tri-, and tetranuclear Co(II) and Cu(II) complexes of three new diSchiff base ligands were synthesized by two different methods. The first method involved the synthesis of the three ligands from condensation reaction of 3,4-dihydroxybenzaldehyde (L′H_2_) with ethylenediamine (en), o-phenylenediamine (o-PD), or 4,5-dimethyl-1,2-phenylendiamine (DMPD) in a mole ratio of 2 : 1 followed by the reaction of the resulting Schiff bases ligands with Cu(II) or Co(II) ions in the presence of 2,2′-bipyridyl (L) to form the di- and trinuclear metal complexes. The second method involved the condensation of the copper complex **LCu(II)L′** (L = 2,2′-bipyridyl, L′ = 4-formylbenzene-1,2-bis(olate)) with en, o-PD, or DMPD in a mole ratio of 2 : 1, respectively, followed by reaction with CuCl_2_ or Cu(ClO_4_)_2_ to form di-, tri-, and tetranuclear copper (II) complexes, respectively. The structures of the ligands and metal complexes were characterized by elemental analyses, NMR, and FTIR spectra. The geometries of metal complexes were suggested according to elemental analysis, electronic spectra, thermal analyses, atomic absorption, and magnetic moments and conductivity measurements.

## 1. Introduction

Multinuclear transition metal complexes have become a central theme of current research because of their potentially useful properties. They are involved in some notable catalytic processes. Their important use for modelling the metal active sites of metalloproteins and their recent applications in the area of nanoscale materials have drawn the focal point of attraction of modern chemists towards the synthesis and characterization of such metal complexes [1]. A number of dinuclear complexes from various types of ligand systems have been prepared and examined in terms of their oxygen uptake or redox processes of oxygen, their catalytic activity, and their antibacterial and antifungal activities [2–4]. One of the synthetic strategies to prepare polynuclear transition metal complexes is the use of simple metal ion complexes which have the appropriate functionality to act as ligands for another metal ion [1]. There is currently a great deal of interest in the synthesis and characterization of polynuclear cobalt, nickel, and copper complexes due to their wide-ranging potential applications such as catalysts, electron transfer mediators in dye-sensitized solar cells, antiviral agents, and molecular nanomagnets [5]. Trinuclear cobalt complexes draw their speciality from their use as catalysts in epoxidation of olefins and in the autoxidation of hydrocarbons [1]. The presence of copper (II) ion in polynuclear complexes has received a wide interest in the fields of (i) the magnetostructural relationship, (ii) the characterization of active sites in multicopper proteins [5], and (iii) biological activity such as antitumor, antiviral, and anti-inflammatory [5]. Schiff bases derived from 3,4-dihydroxybenzaldehyde [3, 4] and diamines like ethylene diamine [5], 2,6-diaminopyridine [2], and 1,4-diaminobenzene [3, 4] represent an important series of chelating agents that have been used to synthesize mono-, di-, or polynuclear transition metal complexes [3–5] in which copper (II) complexes in particular represent models of physical and chemical behavior of biological copper systems that mimic copper metalloproteins such as hemocyanin and tyrosinase [2, 5]. Much attention has been paid to the synthesis and properties of molecules containing the copper (II) complex of 1,10-phenanthroline and 2,2′-bipyridyl units [2, 5–8] which are useful for applications in several fields.

**Figure 1:**
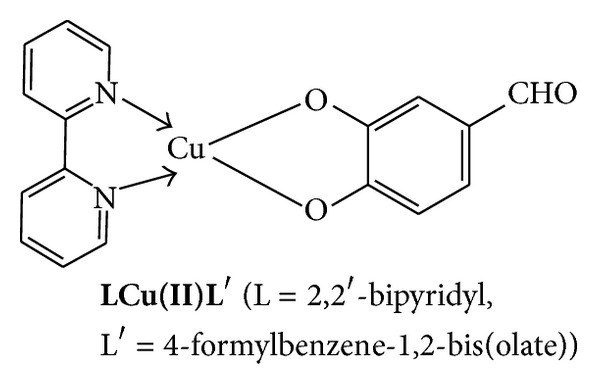


**Figure 2:**
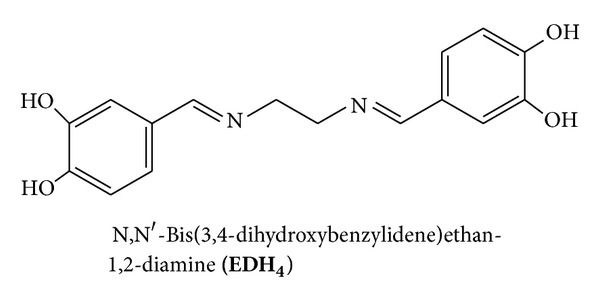


**Figure 3:**
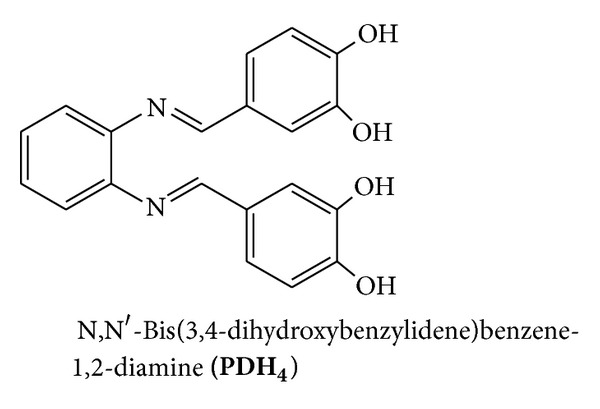


**Figure 4:**
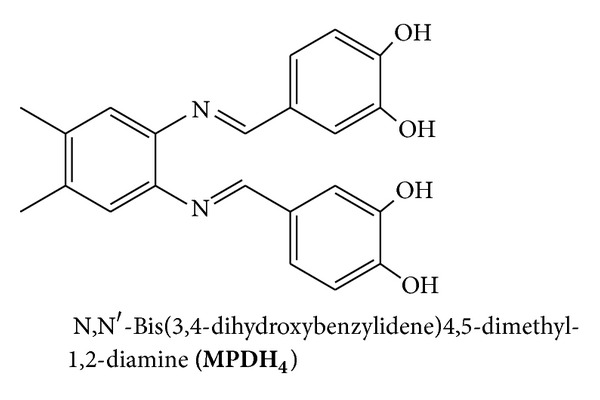


For example, a *π*-conjugated polymer bearing 2,2′-bipyridyl units was found to serve as a unique electrically conducting polymer complex with transition metals such as Ru, Ni, and Fe [6–8]. Nickel (II) mixed ligand complexes involving an aromatic Schiff base and 1,10-phenanthroline showed higher cytotoxic activity than those of the individual ligands [7]. The copper (II) complex of 1,10-phenanthroline was the first synthetic transition metal complex effectively exhibiting nucleolytic activity [5]. In this work, we study for the first time the synthesis of di- and trinuclear copper (II) and cobalt (II) complexes as synthetic models for multicenter active sites of biological systems by following two methods. The first method (method 1) involves the reaction of metal salts with each of the following three new diSchiff base ligands: N,N′-bis(3,4-dihydroxybenzylidene)ethan-1,2-diamine **(EDH**_**4**_ **)** (Figure 2), N,N′-bis(3,4-dihydroxybenzylidene)benzene-1,2-diamine **(PDH**_**4**_ **)** (Figure 3), and N,N′-bis(3,4-dihydroxybenzylidene)-4,5-dimethyl-1,2-diamine **(MPDH**_**4**_ **)** (Figure 4) prepared from the condensation reaction of 3,4-dihydroxybenzaldehyde with ethylenediamine (en), o-phenylenediamine (o-PD), or 4,5-dimethyl-1,2-phenylendiamine (DMPD), respectively. The second method (method 2) involves the condensation reaction of mononuclear copper (II) mixed ligand complex of 3,4-dihydroxybenzaldehyde and 2,2′-bipyridyl (**LCu(II)L**′) (Figure 1) (L = 2,2′-bipyridyl, L′ = 4-formylbenzene-1,2-bis(olate)) with (en), o-PD, or DMPD followed by further reaction with the metal salts to form the tri- and tetrahomonuclear metal complexes.

The structures of the prepared compounds were elucidated depending on elmental analyses, Uv-vis, NMR, and FTIR spectra as well as, thermal analyses, atomic absorption, conductivity measurements, and magnetic susceptibility of metal complexes.

## 2. Experimental

### 2.1. Materials and Methods

All chemicals were of reagent grade and were used as received except o-phenylenediamine and ethanol which were purified and dried as reported previously [9, 10]. Melting points (uncorrected) were determined on Gallenkamp M.F.B 600-010f melting point apparatus.

The elemental analyses were performed on Eurovector EA 3000A. ^1^HNMR and ^13^CNMR were carried out by using Bruker UltraShield 300 MHz NMR spectrophotometer. FTIR spectra were recorded as KBr and CsI discs using Shimadzu FTIR-8400S, Fourier Transform Infrared spectrophotometer. The electronic spectra were recorded in DMF on Shimadzu Uv-visible-160 Spectrophotometer. Thermal analyses (TG & DTG) were carried out under nitrogen atmosphere by using Netzsch Sat 409 PG/PC at a heating rate of 20°C/min under nitrogen atmosphere over a temperature range of 25–1000°C. The metal contents of the complexes were determined by atomic absorption technique using Varian-AA 775 Atomic Absorption spectrophotometer. Electrical conductivity measurements for complexes (10^−3^ M) in DMF at room temperature were carried out by using Hunts Capacitors Trade Mark British made conductivity meter. Magnetic moment (*μ*_eff_ B.M) for the prepared complexes was measured at room temperature by using Bruker Magnet B.M-6.

### 2.2. Preparation Methods

#### 2.2.1. Method 1

*Synthesis of Schiff Bases* ***EDH***_***4***_, ***PDH***_***4***_ *, and* ***MPDH***_***4***_ *: General Procedure.* To a solution of diamine (0.0217, 0.0391, and 0.0493 g for en, O-PD, and DMPD, resp., 0.362 mmol) in a minimum amount of absolute ethanol (en, O-PD) or methanol (DMPD) containing 2 drops of piperidine an ethanolic solution of 3,4-dihydroxybenzaldehyde (0.1 g, 0.724 mmol) was added. Precipitation took place immediately giving yellow, brown, and orange-yellow products, respectively. The mixtures were heated under reflux with continuous stirring for 1 h, 1 h, and 1.5 h, respectively, to allow for complete precipitation. The products were filtered, washed with ethanol, methanol, and ether, and vacuum dried.

*Synthesis of Binuclear (* ***C***_***1***_, ***C***_***2***_ *) and Tetranuclear  (* ***C***_***3***_ *) Copper Bis(bipyridyl) Schiff Base Complexes.* Dinuclear copper complexes **C**_**1**_ and **C**_**2**_ were prepared as follows: to a stirred ethanolic solution of Schiff bases (0.05 g) (0.166 and 0.143 mmol for **EDH**_**4**_ and **PDH**_**4**_, resp.) CuCl_2_·2H_2_O (0.0567 and 0.0489 g, 0.332 and 0.286 mmol, resp.), 2,2′-bipyridyl (0.0519 and 0.0448 g, 0.332 and 0.287 mmol, resp.), and triethylamine (NEt_3_) (0.0673 and 0.0580 g, 0.665 and 0.574 mmol, resp.) were added in a minimum amount of ethanol. Precipitation took place immediately. Reflux was continued for 4 h with continuous stirring. The products were filtered off, washed with ethanol, and vacuum dried. **C**_**3**_ was prepared by treating an ethanolic solution of **MPDH**_**4**_ (0.050 g, 0.132 mmol) with a solution mixture of excess CuCl_2_·2H_2_O (0.100 g, 0.586 mmol), 2,2′-bipyridyl (0.0414 g, 0.265 mmol), and NEt_3_ (0.0537 g, 0.531 mmol) in ethanol. The mixture was heated under reflux for 4 h. A brown precipitate was formed. The product was filtered off, washed several times with hot ethanol, and vacuum dried. 

*Synthesis of a Trinuclear Copper Bis(bipyridyl) Schiff Base Complex (* ***C***_***4***_ *).* To an ethanol solution of **C**_**1**_ (0.05 g, 0.06 mmol) CuCl_2_·2H_2_O (0.0115 g, 0.06 mmol) dissolved in a minimum amount of ethanol was added with continuous stirring for 1 h during which the color of solution changed to dark brown. The mixture was heated under reflux for 4 h. A brown precipitate was formed. The product was filtered off, washed with ethanol, and vacuum dried.

*Synthesis of Trinuclear Copper Tris(bipyridyl) Complexes of* ***EDH***_***4***_ (***C***_***5***_ *) and* ***MPDH***_***4***_ (***C***_***6***_ *).* To a hot solution of **C**_**1**_ (0.05 g, 0.06 mmol) in hot ethanol Cu(ClO_4_)_2_·6H_2_O (0.0222 g, 0.06 mmol) and 2,2-bipyridyl (0.0093 g, 0.06 mmol) in ethanol were added with continuous stirring for 1 h followed by heating under reflux for 4 h to allow for complete precipitation. The resulting product (**C**_**5**_) was filtered off, washed with hot ethanol, and vacuum dried. **C**_**6**_ was prepared by adding a solution of Cu(ClO_4_)_2_·6H_2_O (0.0678 g), 2,2′-bipyridyl (0.0285 g) (0.183 mmol each), and NEt_3_ (0.0246 g, 0.244 mmol) in ethanol to **MPDH**_**4**_ (0.0229 g, 0.061 mmol) dissolved in a minimum amount of ethanol. A dark brown precipitate started to appear. The mixture was heated under reflux for 3-4 h for complete precipitation. The product was filtered, washed with hot ethanol, and vacuum dried.

*Synthesis of Tetra- and Trinuclear Cobalt Bis- and Tris(bipyridyl) Complexes of* ***EDH***_***4***_ *and* ***MPDH***_***4***_ (***C***_***7***_ *and* ***C***_***8***_ *).* **C**_**7**_ was prepared as follows: a solution of 2,2′-bipyridyl (0.0517 g, 0.332 mmol) and NEt_3_ (0.0672 g, 0.665 mmol) in a minimum amount of ethanol was added to a solution of **EDH**_**4**_ (0.0499 g, 0.1664 mmol) in warm ethanol with continuous stirring. Then a solution of CoCl_2_·6H_2_O (0.1592 g, 0.669 mmol) in ethanol was added. The color of solution was changed from blue to brown. The reaction mixture was then heated under reflux for 4 h. A brown precipitate was formed. The product was filtered, washed with hot ethanol, and vacuum dried. The preparation and purification of **C**_**8**_ (dark green) was carried out in the same manner, but the quantities of the reactants were **MPDH**_**4**_ (0.0625 g, 0.1664 mmol), 2,2′-bipyridyl (0,0778 g, 0.4992 mmol), CoCl_2_·6H_2_O (0.1187 g, 0.499 mmol), and NEt_3_ (0.0672 g, 0.665 mmol) and the color of solution after the addition of the cobalt salt was changed from yellow to green.

#### 2.2.2. Method 2

In this method the metal complexes were prepared from condensation reaction of the Cu(II) complex precursor (**LCuL** ^**′**^) (L = 2,2′-bipyridyl, L^**′**^= 4-formylbenzene-1,2-bis(olate)) with the diamines followed by the reaction with the metal salts to form tri- and tetranuclear complexes.

*Synthesis of* ***LCuL*** ^***′***^.  This complex was prepared by following a previously published method [11] with modification. A solution of CuCl_2_·2H_2_O (0.1234 g, 0.724 mmol) in ethanol was added to an ethanolic mixture of 3,4-dihydroxybenzaldehyde (0.1 g, 0.724 mmol), 2,2′-bipyridyl (0.1130 g, 0.724 mmol), and triethylamine (0.1465 g, 1.448 mmol). The reaction mixture was stirred for 20 min. at room temperature during which a brown precipitate was formed. The mixture was heated under reflux for 2 h and the resulting product was separated by filtration, washed with hot ethanol, and dried under vacuum. The product was characterized by elemental analysis and the FTIR spectral analysis.

*Synthesis of Binuclear Copper Bis(bipyridyl) Schiff Base Complexes* ***C***_***9***_, ***C***_***10***_ *, and* ***C***_***11***_. An ethanol solution of diamine (en, O-PD, and DMPD, 0.0140, 0.0151, and 0.0191 g, resp., 0.14 mmol) was added to a solution of **LCuL**′ (0.1 g, 0.28 mmol) in hot ethanol with stirring for 30 min. The mixture was then heated under reflux for 3 h to allow for complete precipitation. The products were filtered off, washed with ethanol and ether, and vacuum dried. 

*Synthesis of Trinuclear Copper Bis(bipyridyl) Schiff Base Complexes* ***C***_***12***_ *and* ***C***_***13***_. To a solution of **C**_**9**_ and **C**_**11**_ (0.0441 and 0.0519 g, resp., 0.06 mmol) in hot ethanol CuCl_2_·2H_2_O (0.0115 g, 0.06 mmol) was added in a minimum amount of ethanol with stirring for 1 h. The colors of solutions changed to brown and precipitation of products took place. The mixtures were heated under reflux for 4 h and the products were filtered off, washed with ethanol, and vacuum dried. 

*Synthesis of Trinuclear Copper Tris(bipyridyl) Schiff Base Complexes* ***C***_***14***_ *and* ***C***_***15***_. A solution mixture of 2,2′-bipyridyl (0.0093 g, 0.06 mmol) and Cu(ClO_4_)_2_·6H_2_O (0.0222 g, 0.06 mmol) in ethanol was added to a solution of **C**_**9**_ and **C**_**10**_ (0.0441 and 0.0536 g, resp., 0.06 mmol) in hot ethanol with stirring for 1 h until the formation of precipitates was observed. The mixtures were then heated under reflux for 4 h and the products were filtered off, washed with hot ethanol, and vacuum dried.

## 3. Results and Discussion

### 3.1. Synthesis

**Scheme 1:**
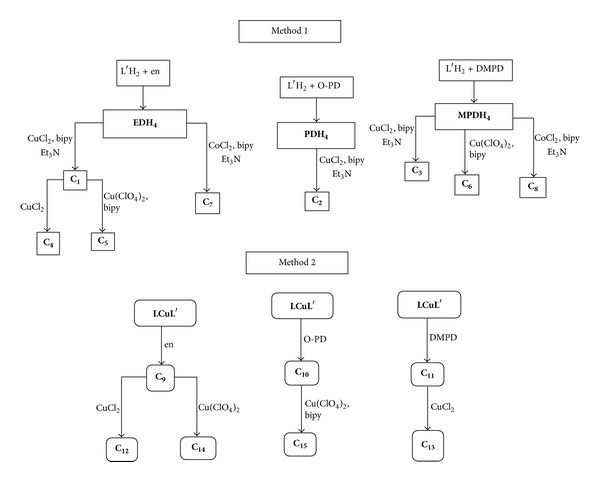
The synthesis routes of the studied metal complexes by two different methods.

The synthesis routes for the metal complexes by the two methods are illustrated in Scheme 1. Method 1 involves the condensation reaction of the diamines with two molecules of 3,4-dihydroxybenzaldehyde (L^**′**^H_2_) to form the diSchiff bases as a first step for the formation of metal complexes. In the second method the condensation reaction occurs between the diamine molecule and two molecules of the mixed ligand copper complex precursor **LCuL** ^**′**^. Although the last method is simpler than the first method to avoid side reactions, the copper complex  precursor is slightly soluble in cold ethanol and therefore reaction with this complex required heating in large amount of solvent.

### 3.2. Physical Properties

The physical properties and results obtained from elemental analyses of the prepared compounds are described in Table 1. The analytical data are quite agreeable with calculated values with few exceptions which were attributed to incomplete combustion of the compounds. The molecular formula of the prepared compounds has been suggested according to the aforementioned data together with those obtained from spectral and thermal analyses as well as conductivity and magnetic susceptibility measurements of metal complexes. All complexes were noncrystalline which made it difficult to obtain their single crystal structures.

### 3.3. Infrared Spectra

The important vibrational modes of IR spectra for the free Schiff bases and their metal complexes are described in Table 2. The spectra of the free ligands displayed strong to moderate absorption band in the wavenumber region 3251–3263 cm^−1^ which were assigned to intramolecular hydrogen bonding of the two adjacent OH groups [12]. These bands were absent in the spectra of all metal complexes which indicates that the phenolic oxygen atoms were bonded to the metal ions [3–5, 11]. The spectrum of the mixed ligand copper (II) complex precursor **CuLL** ^**′**^ displayed strong absorption bands at 1660 and 1540 cm^−1^ assigned to the stretching vibrations of the C=O and C=N groups of L^**′**^ and L moieties, respectively [11, 13]. The low intensity bands observed at lower wavenumber region at 440 and 345 cm^−1^ were assignable to stretching vibrations of Cu–O and Cu–N bonds, respectively [3, 5].

**Figure 5:**
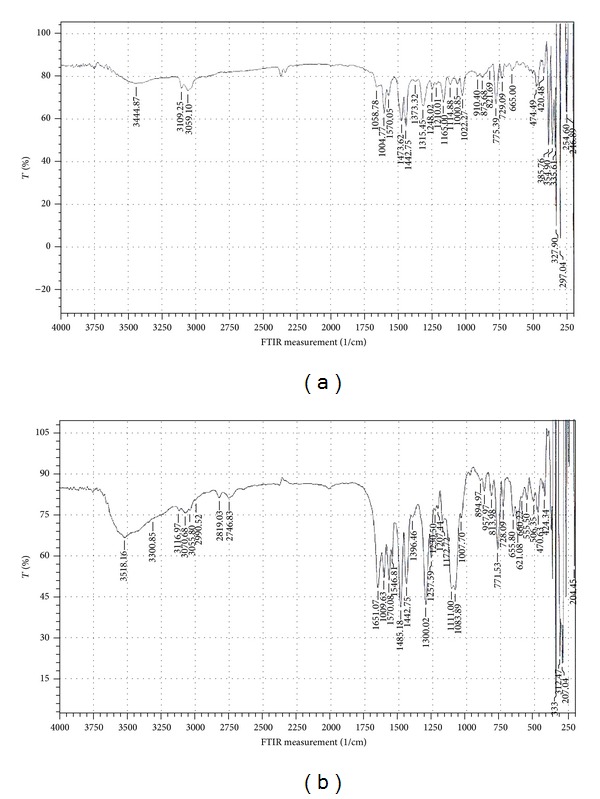
FTIR spectra of (a) **C**_**3**_ and (b) **C**_**5**_.

The strong bands observed at 1631–1670 cm^−1^ and 1604, 1608 cm^−1^ in the spectra of the free Schiff bases were assigned to the asymmetric symmetric stretching vibration of the azomethine group (*ν*C=N) [2–5]. These bands were shifted to lower frequency in all complexes (except **C**_**1**_, **C**_**2**_, **C**_**9**_, **C**_**10**_, and **C**_**11**_) indicating the coordination of the Schiff bases with the metal ions through the azomethine nitrogens [5]. All complexes exhibited strong bands at wavenumber range 1500–1590 cm^−1^ attributed to *ν*_C=N_ of coordinated bipyridyl ligand [14]. The spectra of the complexes **C**_**5**_, **C**_**6**_, **C**_**14**_, and **C**_**15**_ exhibited strong bands assigned to the stretching vibrational modes of ClO_4_ anion (*ν*_ClO_4__) which behaved as monodentate ligand in **C**_**6**_ and **C**_**15**_ complexes [15] and as a free ion in **C**_**5**_ and **C**_**14**_ complexes [15]. The bands appeared at 3400–3750 cm^−1^ in the spectra of **PDH**_**4**_, **C**_**2**_, **C**_**3**_, **C**_**6**_, **C**_**7**_, **C**_**10**_, **C**_**11**_, **C**_**12**_, and **C**_**15**_ were attributed to vibrational modes of lattice H_2_O [15], while the bands which appeared at 3100–3380 cm^−1^ and 621–775 cm^−1^ in the spectra of **C**_**1**_, **C**_**2**_, **C**_**4**_, **C**_**6**_, **C**_**7**_, **C**_**8**_, **C**_**12**_, **C**_**13**_, and **C**_**15**_ were due to coordinated H_**2**_O [15]. The spectrum of **MPDH**_**4**_ exhibited a strong band in the range 3552–3421 cm^−1^ and another band at 1195–1161 cm^−1^ attributed to OH and C–O stretching vibrations of methanol embedded in the crystal lattice of the ligand [13, 15]. Further bands which appeared at lower frequencies in the spectra of metal complexes were assigned to M–O, M–N and M–Cl stretching vibrations (Table 2).  Figure 5 shows representative FTIR spectra of **C**_**3**_ and **C**_**5**_ prepared by method 1 from **MPDH**_**4**_ and **EDH**_**4**_, respectively.

### 3.4. ^1^H NMR and ^13^C NMR Spectra

**Figure 6:**
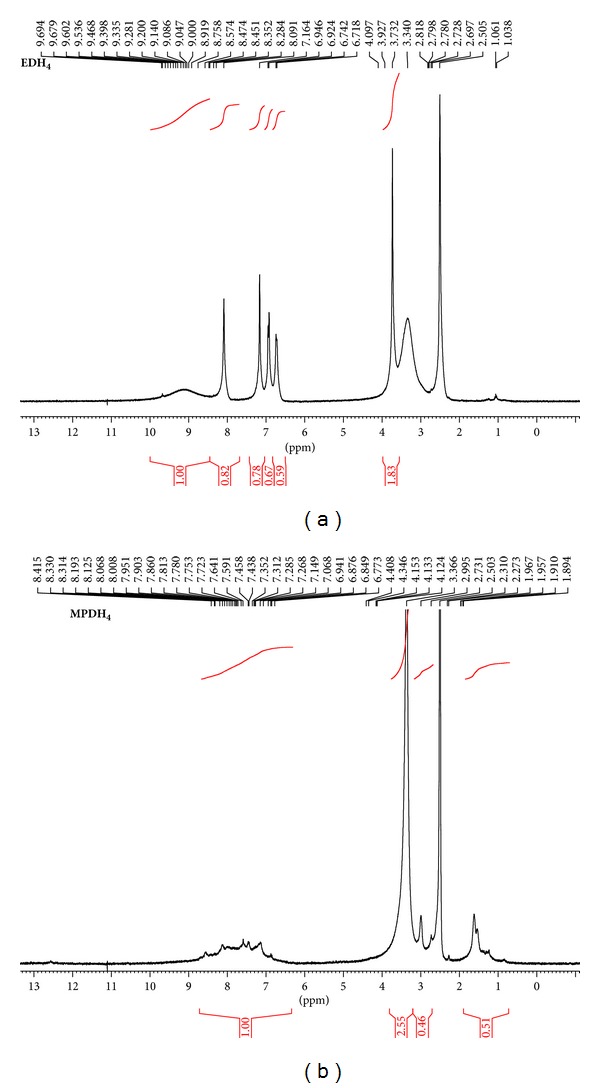
^1^H NMR spectra of diSchiff base ligands **EDH**_**4**_ and **MPDH**_**4**_.

The ^1^H NMR spectra of diSchiff bases and the binuclear bis(bipyridyl) copper complex of **MPDH**_**4**_ (**C**_**11**_) were recorded in DMSO and the chemical shifts and peak assignments are given in Table 3. The spectra of the Schiff base ligands showed a broad peak in the range *δ* = 8.5–9.9 ppm attributed to phenolic hydroxyl protons [3, 4, 16] as is demonstrated by the spectra of **EDH**_**4**_ and **MPDH**_**4**_ shown in Figure 6.

The absence of this peak in the spectrum of complex **C**_**11**_ (Table 3) confirms the involvement of deprotonated hydroxyls in chelation to the metal ion [16, 17]. The peaks displayed by ^1^H NMR spectra of Schiff bases in the range *δ* = 7.9–8.7 ppm were attributed to chemical shifts of the azomethine protons (HC=N) [3, 4, 16, 17]. The spectrum of **C**_**11**_ exhibited the absence of the signals related to OH protons and the appearance of the azomethine proton signals downfield which confirms the formation of the metal complex [16, 17].

Signals of aromatic and aliphatic protons were observed in the chemical shift ranges 6.5–7.9 and 1.5–3.9 ppm, respectively [13]. Chemical shifts for ^13^C NMR of **EDH**_**4**_ and **MPDH**_**4**_ in DMSO are described in Table 4. The signals assigned to the chemical shifts of methylene and methyl groups for the two ligands, respectively, were observed at 60.99 (CH_2_) and at 19.8 and 20.1 (CH_3_) ppm [18], while the signals of aromatic carbons were located at 113.6–161.2 and 110.8–146.8 ppm, respectively [18, 19]. The signals observed at 167.2 and 150.6–152.7 ppm, respectively, were attributed to the chemical shifts of azomethine carbons which confirms the formation of the Schiff bases [20–22].

### 3.5. Electronic Spectra Conductivity and Magnetic Susceptibility Measurements

**Scheme 2:**
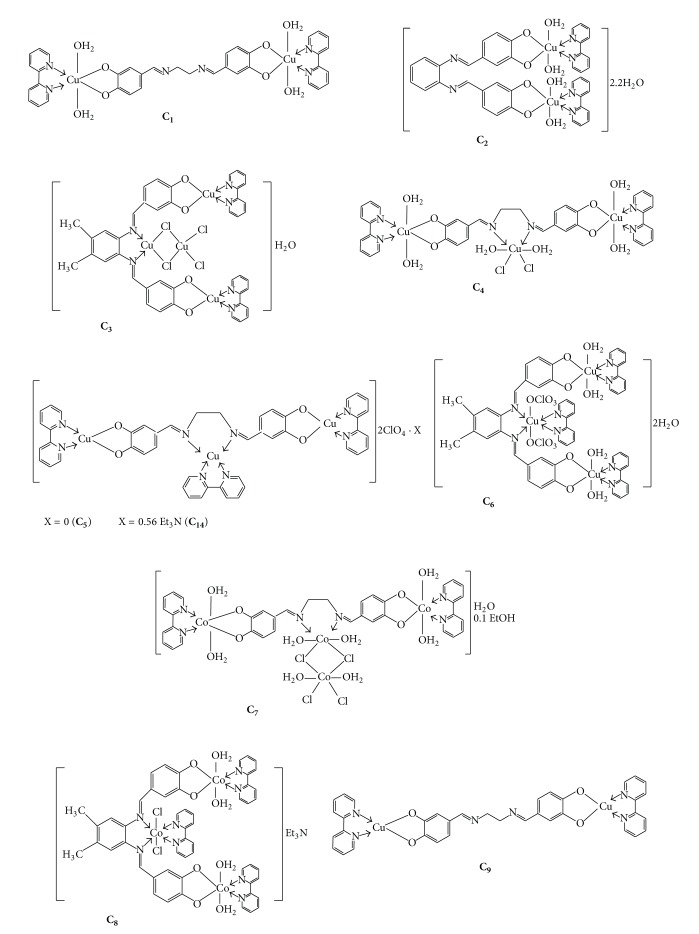

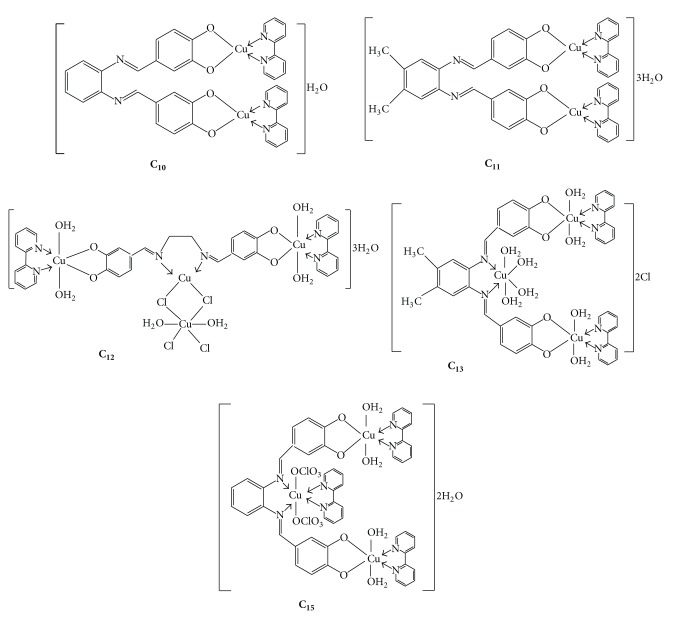
Suggested stereochemical structures of the synthesized diSchiff base complexes.

The results of electronic spectra of the ligands and their metal complexes in DMF are described in Table 5. The three ligands exhibited high intensity bands which appeared at wavenumber region 33333–24390 cm^−1^ and low intensity bands at 27777–20833 cm^−1^ which were assigned to *π* → π* and *n* → π* transitions, respectively [13]. The spectra of metal complexes exhibited hypsochromic shifts of the ligand *π* → π* band which refers to complex formation with the metal ions [18]. The spectra of complexes exhibited additional medium intensity bands in the near Uv to visible region at 28248–23255 cm^−1^ which were attributed to charge transfer transitions [23]. The copper complexes (**C**_**3**_, **C**_**5**_, **C**_**9**_, **C**_**10**_, **C**_**11**_, and **C**_**14**_) displayed bands in the regions 12970–10298 cm^−1^, 18833–15313 cm^−1^, and 21739–19920 cm^−1^ assigned to ^**2**^B_**1g**_ **→** ^**2**^A_**1g**_, ^**2**^B_**1g**_ **→** ^**2**^B_**2g**_, and ^**2**^B_**1g**_ **→** ^**2**^E_**g**_ transitions, respectively, of square planar Cu(II) complexes [23–27] while the spectra of the copper complexes (**C**_**1**_, **C**_**2**_, **C**_**4**_, **C**_**6**_, **C**_**12**_, **C**_**13**_, and **C**_**15**_) displayed bands in the regions 13513–10460, 18868–15431, and 23148–22472 which were attributed to the transitions of tetragonally distorted octahedral Cu(II) complexes [23–25]. The two cobalt complexes (**C**_**7**_ and **C**_**8**_) exhibited two bands observed at 15731 and 15983 cm^−1^, respectively, which were assigned to ^**4**^T_**1g**_ **→** ^**4**^T_**1g**_ (P) (*ν*_3_) and at 10504 and 9900 cm^−1^, respectively, corresponding to the transition ^**4**^T_**1g**_ **→** ^**4**^A_**2g**_ (*ν*_2_) of octahedral Co(II) complexes [23]. The energies of *ν*_1_ (^**4**^T_**1g**_ **→** ^**4**^T_**2g**_) as well as the values of the spectral parameters Dq/B^**′**^, B^**′**^, 10Dq, and nephelauxetic ratio *β* for the Co(II) complexes **C**_**7**_ (7083 cm^−1^, 0.9, 787 cm^−1^, 7080 cm^−1^, and 0.811, resp.), and **C**_**8**_ (6456 cm^−1^, 0.7, 807 cm^−1^, 5740 cm^−1^, and 0.831, resp.), were calculated by applying the band ratio *ν*_3_/*ν*_2_ on Tanabe-Sugano diagram of d^7^ complexes. The values of *β* indicate a covalent bonding character of both complexes [23]. Conductivity measurements in DMF showed nonelectrolytic nature for all compounds (Table 3) except **C**_**5**_, **C**_**13**_, and **C**_**14**_ which were electrolytes with ionic ratio (1 : 2) [28]. Magnetic susceptibility measurements at room temperature showed that the magnetic moment (*μ*_eff_) of the Cu(II) complex precursor **CuLL** ^**′**^ (1.69 B.M) agrees with square planar geometry of the complex [11, 23]. The values of *μ*_eff_ of the other complexes were less than those expected for copper and cobalt ions which imply that the di- and trinuclear copper (II) and cobalt (II) complexes possess antiferromagnetic properties by a strong intramolecular antiferromagnetic spin exchange interaction [5]. According to the aforementioned results in addition to elemental analysis and FTIR and NMR spectra the stereochemical structures of the studied complexes were suggested as is illustrated in Scheme 2.

### 3.6. Thermal Analysis

**Figure 7:**
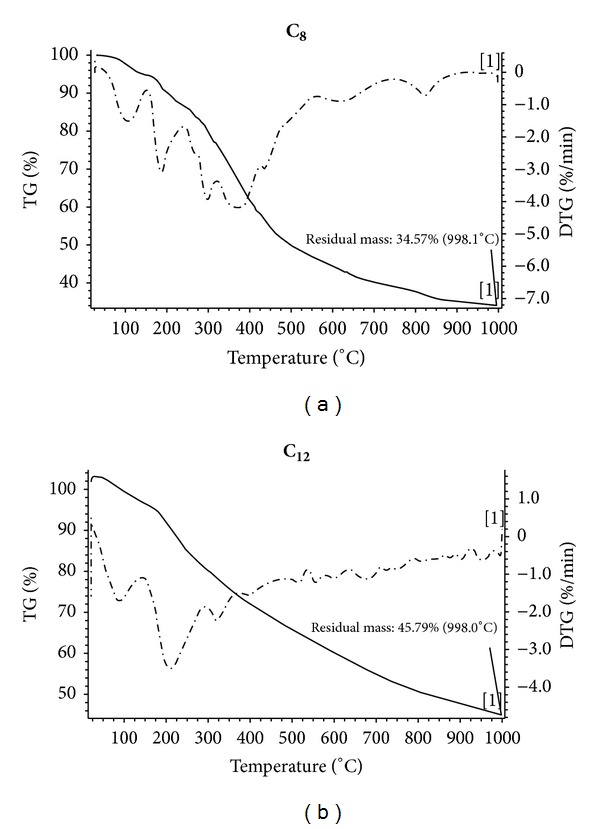
TG and DTG thermographs of **C**_**8**_ and **C**_**12**_.

Thermogravimetric (TG) and differential thermogravimetric (DTG) analyses for the two complexes **C**_**8**_ and **C**_**12**_ are shown in Figure 7. The decomposition temperature and the weight losses are described in Table 6. The loss of solvent molecules embedded in the crystal lattice of the complexes as well as uncoordinated ligand groups took place at the first stage at temperature range 78–180°C with peak temperatures at 100 and 98°C, respectively, as is indicated by the DTG curves of the two complexes (Figure 7). The successive loss of coordinated water molecules occurred in the second and third stages at peak temperatures 195, 300 and 210, 325°C, respectively. The loss of bipyridyl and chloride ligands took place at temperature range 400–900°C. The DTG curve of **C**_**8**_ showed three peaks at 412, 620, and 822°C for this stage. The high percentage of the remaining residues at 1000°C indicates that the two complexes are very stable and require a higher temperature range for complete decomposition which is quite common for polynuclear metal complexes [5].

## 4. Conclusions

The bi-, tri-, and tetranuclear bis- and tris(bipyridyl) copper (II) and cobalt (II) mixed ligand complexes of three diSchiff base ligands derived from 3,4-dihydroxybenzaldehyde and three diamines in a stoichiometric ratio of 2 : 1 were successfully synthesized by two different methods. The structures of the ligands were confirmed by elemental and spectral analysis. Coordination of the metal ions to form trinuclear and tetranuclear complexes took place through the two imino nitrogens and phenolic dianionic oxygen atoms of each ligand molecule as was indicated by FTIR spectra. The formation of binuclear diSchiff base copper bis(bipyridyl) complexes was achieved by reacting the mononuclear copper (II) mixed ligand complex **CuLL** ^**′**^ with the diamines in a 2 : 1 ratio, respectively, as was confirmed by the NMR spectrum of **C**_**11**_ while trinuclear bis- and tris(bipyridyl) and tetranuclear complexes of the three ligands were achieved by reacting the synthesized Schiff bases with the copper salts in the presence of 2,2′-bipyridyl. The complexes exhibited low values of magnetic moments which made them a good synthetic model for intramolecular antiferromagnetic spin exchange interaction of biological systems. In the future work the enzyme like and metalloprotein activities of these complexes and their biological activities will be studied in detail.

## Figures and Tables

**Table 1:** The physical properties and analytical data for Schiff bases and their metal complexes.

Symbol	Color	(m.p.) °C	Yield %	CHN % analysis found (Calc.)			M % found (Calc.)	Cl % found (Calc.)
				C %	H %	N %		
**EDH_4_**	Yellow	210 dec.	67.07	65.04 (64.00)	5.77 (5.33)	9.78 (9.33)	—	—
**PDH_4_·4H_2_O**	Dark brown	240	30.03	57.78 (57.14)	3.84 (4.28)	7.32 (6.66)	—	—
**MPDH_4_·MeOH**	Pale yellow	222	56.82	67.50 (67.60)	6.24 (5.88)	7.72 (6.86)	—	—
**C_1_**	Brown	>280	68.92	53.29 (53.52)	4.31 (4.46)	10.39 (10.40)	16.05 (15.74)	—
**C_2_**	Dark brown	>280	37.55	52.77 (53.65)	4.46 (4.51)	8.92 (9.38)	14.95 (14.20)	—
**C_3_**	Brown	>280	62.24	46.00 (45.89)	3.79 (3.09)	8.25 (7.64)	23.79 (23.14)	12.57 (12.93)
**C_4_**	Dark brown	>280	67.53	44.67 (44.18)	3.78 (4.09)	7.80 (8.59)	19.92 (19.50)	7.47 (7.26)
**C_5_**	Dark brown	>280	76.95	47.77 (47.84)	3.90 (3.12)	10.14 (9.71)	16.00 (16.52)	5.55 (6.15)
**C_6_**	Dark brown	>280	63.57	46.23 (46.65)	3.82 (3.88)	9.13 (8.37)	14.98 (14.25)	5.82 (5.31)
**C_7_**	Dark brown	>280	26.10	37.78 (37.70)	4.22 (4.04)	6.85 (7.29)	20.85 (20.44)	12.11 (12.32)
**C_8_**	Dark green	>280	26.68	55.44 (55.20)	5.32 (4.99)	9.90 (9.99)	13.74 (14.02)	6.03 (5.63)
**C_9_**	Dark brown	>280	68.06	59.50 (58.76)	4.55 (3.81)	11.73 (11.42)	17.93 (17.28)	—
**C_10_**	Dark brown	>280	86.26	59.38 (59.91)	4.15 (3.74)	9.61 (10.48)	16.54 (16.22)	—
**C_11_**	Brown	>280	77.00	58.39 (58.26)	4.12 (4.39)	8.92 (9.71)	15.22 (14.69)	—
**C_12_**	Dark brown	>280	52.56	36.24 (37.04)	4.02 (3.43)	7.9 (7.20)	22.42 (21.79)	11.59 (12.17)
**C_13_**	Brown	>280	57.05	45.80 (46.25)	4.12 (4.40)	8.33 (7.70)	16.47 (17.49)	7.08 (6.51)
**C_14_**	Reddish brown	>280	93.03	51.24 (50.64)	3.90 (4.52)	10.53 (10.20)	15.23 (14.54)	5.14 (5.41)
**C_15_**	Brown	>280	65.50	45.18 (45.81)	3.96 (3.66)	7.93 (8.55)	13.91 (14.55)	6.05 (6.07)

**Table 2:** Significant bands in the FTIR spectra (cm^−1^) for Schiff bases and their metal complexes.

Symbol	ν_OH_	ν_C−H_	ν_C=N_ imine	ν_C=N_ bipy.	ν_ClO4_ ionic (Coord.)	ν H_2_O lattice (Coord.)	ν_M−O_	ν_M−N_	ν_M−Cl_
**EDH_4_**	3263	2839	1651	—	—	—	—	—	—
			1608						
**PDH_4_·4H_2_O**	3253	2750	1631	—	—	3448–3417	—	—	—
			1604						
**MPDH_4_·MeOH**	3251	2985	1670	—	—	—	—	—	—
**C_1_**	—	2860	1654	1519	—	(3150, 767, 651)	420	370	—
		2760	1608						
**C_2_**	—	2950	1640	1500	—	3400 (3250, 775, 660)	460	350	—
			1610						
**C_3_**	—	2900	1658	1570	—	3444	474	385	297^a^
			1604						254^b^
**C_4_**	—	2800	1643	1543	—	(3356, 771, 729)	470	385	340^a^
		2750	1604						
**C_5_**	—	2819	1651	1570	1111, 1083, 1037	—	470	333	—
		2746	1608						
**C_6_**	—	2950	1660	1570	(1093, 1040)	3580 (3240, 750, 675)	550	341	—
		2800	1610						
**C_7_**	—	2750	1653	1580	—	3750 (3300, 770, 650)	490	405	312^a^
			1610						241^b^
**C_8_**	—	2980	1640	1560	—	(3240, 775, 655)	560	395	325^a^
		2870	1610						270^b^
**C_9_**	—	2951	1655	1573	—	—	478	358	—
		2839							
**C_10_**	—	2980	1630	1590	—	3550	490	400	—
**C_11_**	—	2823	1651	1570	—	3456	489	389	—
		2754	1608						
**C_12_**	—	2950	1645	1550	—	3700 (3250, 690, 640)	450	322	304^a^
		2800	1600						250^b^
**C_13_**	—	2960	1660	1550	—	(3300, 770, 640)	560	343
		2850	1610					
**C_14_**	—	2750	1639	1570	1103, 1050	—	459	393
			1604					
**C_15_**	—	2800	1620	1550	(1091, 1050)	3600 (3380, 771, 740)	510	400	

^a^Terminal; ^b^bridged.

**Table 3:** ^
1^H NMR data of the three Schiff base ligands and the Cu(II) complex **C_11_** in DMSO.

**EDH_4_**		**PDH_4_**	
Chemical shift *δ* (ppm)	Assignments	Chemical shifts *δ* (ppm)	Assignments
(8.8–9.9, 4H, b)	Protons of OH	(9.60–9.75, 4H, b)	Protons of OH
(8.1, 2H, s)	Protons of azomethine	(8.7–7.9, 2H, b)	Protons of azomethine
(6.7–7.4, 6H, m)	Aromatic protons	(6.5–7.9, 10H, b)	Aromatic protons
(3.9, 4H, s)	Protons of NCH_2_	(3–3.5, 8H, b)	Protons of H_2_O
(3–3.5, 2H, b)	Protons of H_2_O (in DMSO)	(2.5, 6H, s)	Protons of DMSO
(2.5, 6H, s)	Protons of DMSO		

**MPDH_4_**		**C_11_ complex**	
Chemical shifts *δ* (ppm)	Assignments	Chemical shifts *δ* (ppm)	Assignments

(8.5–8.68, 4H, b)	Protons of OH	(10.1–10.98, 2H, b)	Protons of azomethine
(8.0–8.28, 2H, b)	Protons of azomethine	(6.7–7.5, 24H, b)	Aromatic protons of benzene rings and bipyridyl
(6.9–7.6, 10, m)	Aromatic protons	(3.2, 6H, m)	Protons of H_2_O
(3.0–3.6, 2H, m)	Protons of H_2_O	(2.4–2.8, 6H, m)	Protons of DMSO
(2.5, 6H, s)	Protons of DMSO	(0.97–1.85, 6H, b)	Protons of CH_3_
(1.5–1.7, 6H, m)	Protons of CH_3_	—	—

**Table 4:** Chemical shifts (ppm) for ^13^C NMR of Schiff bases **EDH_4_** and **MPDH_4_** in DMSO.

Compound	Chemical shifts *δ* (ppm)	Assignments
**EDH_4_**	60.99	Carbon of CH_2_
	113.6–161.2	Aromatic carbons
	167.2	Carbon of HC=N

**MPDH_4_**	19.8, 20.1	Carbon of methyl group
	110.8–146.8	Aromatic carbons
	150.6, 152.7	Carbon of HC=N

**Table 5:** Electronic spectra, magnetic moments, and molar conductivity data of Schiff bases and their metal complexes.

Symbol	Band positions (cm^−1^)	Assignment	*μ*_eff_ (B.M)	Molar conductivity S·mol^−1^·cm^2^ in DMF
**EDH_4_**	33222, 24691	*π* → π*	—	0.0018
**PDH_4_**	33222, 27777	*π* → π*	—	0.011
	20833	*n* → π*		
**MPDH_4_**	33333	*π* → π*	—	0.0007
	27777	*n* → π*		
**C_1_**	32362, 29154	Intraligand *π* → π*	0.386 oh	0.022
	23255	C.T		
	15431	^ 2^B_1_g → ^2^B_2_g		
	13513	^ 2^B_1_g → ^2^A_1_g		
**C_2_**	33444	Intraligand *π* → π*	0.514 oh	0.251
	26315	C.T		
	18868	^ 2^B_1_g → ^2^B_2_g		
**C_3_**	33333	Intraligand *π* → π*	1.260 Sq.	0.093
	25641	C.T		
	15625	^ 2^B_1_g → ^2^B_2_g		
	12658	^ 2^B_1_g → ^2^A_1_g		
**C_4_**	37735, 34843	Intraligand *π* → π*	1.023 oh	0.046
	27247	C.T		
	23148	^ 2^B_1_g → ^2^Eg		
	10989	^ 2^B_1_g → ^2^A_1_g		
**C_5_**	34013, 27247	Intraligand *π* → π*	1.061 Sq.	149
	23313	C.T		
	16025	^ 2^B_1_g → ^2^B_2_g		
**C_6_**	34129, 28571	Intraligand *π* → π*	0.810 oh	0.114
	25974	C.T		
	22883	^ 2^B_1_g → ^2^Eg		
	10460	^ 2^B_1_g → ^2^A_1_g		
**C_7_**	36363, 33333	Intraligand *π* → π*	2.440 oh	0.043
	31250			
	26178	C.T		
	15731	^ 4^T_1_g → ^4^T_1_g (P)		
	10504	^ 4^T_1_g → ^4^A_2_g		
	7083 cal.	^ 4^T_1_g → ^4^T_2_g		
**C_8_**	34843, 33003	Intraligand *π* → π*	0.200 oh	0.273
	28248	C.T		
	15983.	^ 4^T_1_g → ^4^T_1_g (P)		
	9900	^ 4^T_1_g → ^4^A_2_g		
	6456 cal.	^ 4^T_1_g → ^4^T_2_g		
**C_9_**	36363, 27247	Intraligand *π* → π*	1.092 Sq.	0.0002
	23310	C.T		
	19920	^ 2^B_1_g → ^2^Eg		
	15313	^ 2^B_1_g → ^2^B_2_g		
	10298	^ 2^B_1_g → ^2^A_1_g		
**C_11_**	29585	Intraligand *π* → π*	2.017 Sq.	0.005
	26041	C.T		
	21739	^ 2^B_1_g → ^2^Eg		
	15873	^ 2^B_1_g → ^2^B_2_g		
**C_12_**	36496, 32894	Intraligand *π* → π*	1.023 oh	0.013
	27624	C.T		
	22935	^ 2^B_1_g → ^2^Eg		
	10482	^ 2^B_1_g → ^2^A_1_g		
**C_13_**	33333, 29239	Intraligand *π* → π*	0.440	163
	27027	C.T		
	22472	^ 2^B_1_g → ^2^Eg		
**C_14_**	34013, 29154	Intraligand *π* → π*	1.89 Sq.	158
	27027	C.T		
	18833.	^ 2^B_1_g → ^2^B_2_g		
**C_15_**	29325	Intraligand *π* → π*	1.783 oh	0.023
	27397	C.T		
	22727	^ 2^B_1_g → ^2^Eg		

**Table 6:** Thermal decomposition of the copper complexes (**C_8_** and **C_12_**).

**C_8_** stable phase [MPD(Co_3_(bipy)_3_(H_2_O)_4_Cl_2_] Et_3_N M.wt = 1260.7	Temp. range of decomp. °C	Weight % loss found (calc.)
↓ 0.45Et_3_N + Cl + 2H_2_O	78–211	9.00 (9.28)
↓ 0.55Et_3_N + 2H_2_O	212–423	6.80 (7.26)
↓ 2CH_3_ + 2bipy + C_6_H_2_	424–661	32.4 (33.43)
↓ Cl + bipy + HCN	662–998	17.23 (17.33)
C_7_H_4_NO_2_Co_2 _+ C_6_H_4_O_2_Co (residue)	—	34.57 (33.13)

**C_12_** stable phase [ED(Cu_4_(bipy)_2_(H_2_O)_6_Cl_4_] 3H_2_O M.wt = 1166.16	Temp. range of decomp. °C	Weight % loss found (calc.)

↓ 4H_2_O	73–261	5.80 (6.17)
↓ 5H_2_O + C_2_H_4_	262–365	10.20 (10.11)
↓ C_5_H_4_N + bipy	366–542	20.00 (19.46)
↓ 4Cl	543–761	11.8 (12.13)
↓ C_5_H_4_N	762–998	6.4 (6.68)
C_14_H_8_N_2_O_4_Cu_4_ (residue)	—	45.79 (44.76)

## References

[1] Pal S, Barik AK, Gupta S (2008). Anion dependent formation of linear trinuclear mixed valance cobalt(III/II/III) complexes and mononuclear cobalt(III) complexes of a pyrazole derived ligand—synthesis, characterization and X-ray structures. *Polyhedron*.

[2] Karaböcek N, Karaböcek S, Kormali F (2007). Mono-, di- and trinuclear copper(II) complexes of a Schiff base ligand, 2-{(E)-[(6-{[(1E)-(2-hydroxyphenyl)methylene]amino}pyridin-2-yl)imino]-methyl} phenol. *Turkish Journal of Chemistry*.

[3] Nassar AM, Hassan AM, Elkmasha AN, Ahmed YZ (2012). Synthesis and characterization of novel binuclear complexes. *International Journal of Chemical and Biochemical Sciences*.

[4] Hassan AM, Nassar AM, Ahmed YZ, Elkmash AN (2012). Synthesis, characterization and biological evaluation, of binuclear OF Fe(III), Co(II), Ni(II), Cu(II) and Zn(II) complexes with schiff base (E)-4-[(hydroxyl phenylimino)methyl]benzene-1, 2-diol. *International Journal of Pharmaceutical Sciences and Research*.

[5] Dede B, Karipcin F, Cengiz M (2009). Synthesis, characterization and extraction studies of N,N^*″*^- Bis[1-biphenyl-2-hydroxyimino-2-(4-acetylanilino)-1-ethylidene]-diamines and their homo-and heteronuclear copper(II) complexes. *Journal of Chemical Sciences*.

[6] Lee C, Jeong N (2002). Synthesis of novel 3, 3′-dimethyl-2, 2′-bipyridyl derivatives having nematic liquid-crystalline and photoluminescent properties. *Journal of Industrial and Engineering Chemistry*.

[7] Prashanthi Y, Kiranmai K, Ira, Sathish kumar K, Chityala VK, Shivaraj (2012). Spectroscopic characterization and biological activity of mixed ligand complexes of Ni(II) with 1, 10-phenanthroline and heterocyclic schiff bases. *Bioinorganic Chemistry and Applications*.

[8] Yildiz SZ, Misir MN, Tüfekçi N, Gök Y (1998). The synthesis and characterization of a novel (E,E)-dioxime and its mono- and polynuclear complexes. *Acta Chemica Scandinavica*.

[9] Vogel IA (1972). *Practical Organic Chemistry Qualitative Organic Analysis*.

[10] Perrin DD, Armarego WLF (1980). *Purification of Laboratory Chemicals*.

[11] Patel KV, Bhattacharya PK (1986). Study of some trinuclear copper(II) complexes involving catechol aldehyde and heteroaromatic nitrogen bases. *Polyhedron*.

[12] Fotouhi L, Asadi S, Tammari E, Heravi MM, Nematollahi D (2008). Electrochemical oxidation of catechol and 4-tert-butylcatechol in the presence of 1-methyl-1H-imidazole-2-thiol: synthesis and kinetic study. *Journal of the Iranian Chemical Society*.

[13] Silverstein RM, Webster FX (1997). *Spectrometric Identification of Organic Compounds*.

[14] Bayari S, Ataç A, Yurdakul Ş (2003). Coordination behaviour of nicotinamide: an infrared spectroscopic study. *Journal of Molecular Structure*.

[15] Nakamoto K (1997). *Infrared and Ramman Spectra of Inorganic and Coordination Compounds*.

[16] Chavan VL, Mehta BH (2011). X-ray, thermal and biological studies of Ru(III), Rh(III) and Pd(II) schiff base metal complexes. *Research Journal of Chemistry and Environment*.

[17] Neelakantan MA, Esakkiammal M, Mariappan SS, Dharmaraja J, Jeyakumar T (2010). Synthesis, characterization and biocidal activities of some schiff base metal complexes. *Indian Journal of Pharmaceutical Sciences*.

[18] Tumer M, Deligonul N, Golcu A, Akgun E, Dolaz M (2006). Mixed-ligand copper (II) complexes: investigation of their spectroscopic, catalysis, antimicrobial and potentiometric properties. *Transition Metal Chemistry*.

[19] Mohammed IA, Hamidi RM (2012). Synthesis of new liquid crystalline diglycidyl ethers. *Molecules*.

[20] Mounika K, Anupama B, Pragathi A, Gyanakumari C (2010). Synthesis and characterization and biological activity of schiff base derived from 3-ethoxy salicylaldehyde and 2-amino benzoic acid and its transition metal complexes. * Journal of Scientific Research*.

[21] Ha S-T, Win Y-F, Koh T-M, Chong Y-T (2011). Synthesis and characterization of 4-{[(3-cyanophenyl)imino]methyl}-3-hydroxyphenyl octadecanoate. *Australian Journal of Basic and Applied Sciences*.

[22] Sabic AE, Karabork M, Ceyhan G, Tumer M, Digrak M (2012). Polydentate schiff base ligands and their la(III) complexes: synthesis, characterization, antibacterial, thermal and electrochemical properties. *International Journal of Inorganic Chemistry*.

[23] Lever ABP (1968). *Inorganic Electronic Spectroscopy*.

[24] Angelusiu MV, Almajan GL, Ilies DC, Rosu T, Negoiu M (2008). Cu(II) complexes with nitrogen-oxygen donor ligands: synthesis and biological activity. *Chem. Bull. “POLITEHNICA” University of Timisoara*.

[25] Reddy PM, Rohini R, Krishna ER, Hu A, Ravinder V (2012). Synthesis, spectral and antibacterial studies of copper(II) tetraaza macrocyclic complexes. *International Journal of Molecular Sciences*.

[26] Al-Jeboori MJ, Abdul-Ghani AJ, Al-Karawi AJ (2008). Synthesis and structural studies of new mannich base ligands and their metal complexes. *Transition Metal Chemistry*.

[27] Khaleel AMN, Abdul-Ghani AJ (2009). Synthesis and characterization of new schiff bases derived from N (1)-substituted isatin with dithiooxamide and their co(II), Ni(II), Cu(II), Pd(II), and Pt(IV) complexes. *Bioinorganic Chemistry and Applications*.

[28] Geary WJ (1971). The use of conductivity measurements in organic solvents for the characterisation of coordination compounds. *Coordination Chemistry Reviews*.

